# Peri-implant fracture: a rare complication after intramedullary fixation of trochanteric femoral fracture

**DOI:** 10.1007/s00402-021-04193-4

**Published:** 2021-10-07

**Authors:** Lauri M. Halonen, Antti Stenroos, Henri Vasara, Jussi Kosola

**Affiliations:** 1 Department of Orthopedics and Traumatology, South Karelia Central Hospital, University of Helsinki, Valto Käkelän katu 3, 53130 Lappeenranta, Finland; 2 Department of Orthopedics and Traumatology, Helsinki University Hospital, University of Helsinki, Helsinki, Finland; 3 Department of Orthopedics and Traumatology, Kanta-Häme Central Hospital, Hämeenlinna, Finland

**Keywords:** Hip fracture, Trochanteric fracture, Peri-implant fracture, Complication

## Abstract

**Introduction:**

Trochanteric femoral fractures are among the most common operatively treated fractures. Intramedullary fixation has become the treatment of choice in many centers around the world. Nevertheless, the knowledge of rare complications of these fractures is limited. In this study, the incidence and treatment strategies for peri-implant fractures (PIF) were assessed.

**Materials and methods:**

A single-center retrospective cohort study was done on 987 consecutive operatively treated trochanteric fractures. PFNA cephalomedullary nail was used as a fixation method. All patients were followed up from patient records for peri-implant fractures. Plain radiographs as well as different salvage methods were analyzed and compared.

**Results:**

The total rate of peri-implant fractures was 1.4% (*n* = 14). The rate of PIF for patients treated with short (200 mm) nails, intermediate-length (240 mm) nails, and long nails was 2.7% (*n* = 2), 1.5% (*n* = 11), and 0.7% (*n* = 1), respectively (ns, *p* > 0.05 for difference). Treatment of choice for PIF was either ORIF with locking plate (57%, *n* = 8) or exchange nailing (43%, *n* = 6). None of the PIF patients needed additional surgeries for non-union, malunion, or delayed union.

**Conclusions:**

A PIF is a rare complication of intramedullary fixation of trochanteric fractures. It can be treated with either locking plates or exchange nailing with sufficient results. There are no grounds for favoring long nails to avoid PIFs.

## Introduction

Trochanteric femoral fractures (AO 31-A) are one of the most common operatively treated fractures [1]. Hip fracture patients are usually elderly with multiple comorbidities, which presents a challenge for treatment. The treatment aims to restore mobility, and prevent institutionalization and increased mortality while avoiding reoperations and readmissions. Despite the high prevalence, there is still lacking evidence on the optimal treatment for these fractures [2–5]. An increasing trend toward the use of intramedullary nailing compared to sliding hip screws has been described [6].

With the increasing use of intramedullary nailing, problems regarding peri-implant fractures (PIFs) have been brought up. Although reported incidence for PIFs has been as high as 2.6% [7], no distinct guidelines have been established for the treatment of PIFs. Previous studies do not report outcomes of salvage operations done after PIF.

Previous reports suggest a high overall complication rate, where non-surgical complications such as delirium, anemia, electrolyte disturbances, pulmonary complications, and heart complications form the majority of complications [8, 9]. The most common reasons for reoperation after intramedullary fixation are surgical site infection, mal/non-union, and mechanical complications [10].

The aim of the study was to describe the incidence of a peri-implant fracture (PIF), compare the risk with short and long cephalomedullary nails, and describe the treatment and the treatment results of PIF.

## Patients and methods

The study was a single-center retrospective analysis of 987 consecutive trochanteric fractures on 966 patients (AO/OTA 31-A), all treated with intramedullary nailing with Proximal Femoral Nail Antirotation (PFNA) intramedullary nail (DePuy Synthes, Raynham, Massachusetts, US). A short nail (200 mm) was used in 7.5% (*n* = 74), intermediate-length nail (240 mm) 74% (*n* = 728), and a long nail (300–420 mm) in 15% (*n* = 150) of the fractures. For 3.5% (*n* = 35), nail length was not defined in the charts, but could be deduced to be 200 mm or 240 mm. The patients were operated on in the years 2011–2016 and the follow-up from the patient records was for a minimum of 2 years after the operation or until death.

Initial operations were performed on a traction table according to AO principles [11] under fluoroscopy guidance. Open reduction was performed if sufficient reduction was not achieved with closed reduction methods. The nail length was defined according to the surgeon’s preference. In general, short nail was used for stable trochanteric fractures, intermediate-length or long nail was used for unstable trochanteric fractures, and long nail for subtrochanteric fractures. Distal locking screws were used in all patients. Cement augmentation was not used in any of the cases.

Patient database and radiological records were searched for each patient and reoperations for PIF or nail breakage were analyzed. We collected the patient demographic data, delay to surgery, and comorbidities (ASA class, use of anticoagulants, and other illnesses) (Table 1).

**Table 1:** Primary hip fracture patient characteristics

	PIF (*n* = 14)	No PIF (*n* = 972)	*p* value
Age, years (SD)	80.5 (12.1)	84.8 (11.8)	0.18
Female sex, *n* (%)	10 (71.4%)	670 (68.3%)	0.80
ASA^a^ (SD)	3.00 (0.45)	3.23 (0.63)	0.23
CCI^b^ (SD)	4.93 (1.64)	4.81 (1.81)	0.81
Delay to surgery, days (SD)	2.14 (0.77)	2.19 (0.94)	0.86
Length of stay, days (SD)	6.64 (2.85)	7.17 (3.14)	0.53
Active cigarette smoking, *n* (%)	2 (14.3%)	98 (10.0%)	0.60
Anticoagulation medication, *n* (%)	2 (14.3%)	182 (18.6%)	0.68
3-month mortality, n (%)	0 (0.0%)	138 (14.1%)	0.13
2-year mortality, *n* (%)	4 (28.6%)	349 (35.6%)	0.59

Given values are means if not otherwise specified

^a^American Society of Anesthesiologists classification

^b^Charlson Comorbidity Index

The differences in demographic and preoperative characteristics between the groups were tested using the Chi-square test or Student t test when appropriate. *p* values of < 0.05 were considered significant. The statistical program SPSS 25 (IBM Corp. released 2017. Armonk, NY: IBM Corp.) was used for analyzes.

The research was approved by the research committee in Helsinki University Hospital. As the study was a retrospective chart review without interaction with the patients, an ethical committee approval was not sought.

## Results

**Fig. 1:**
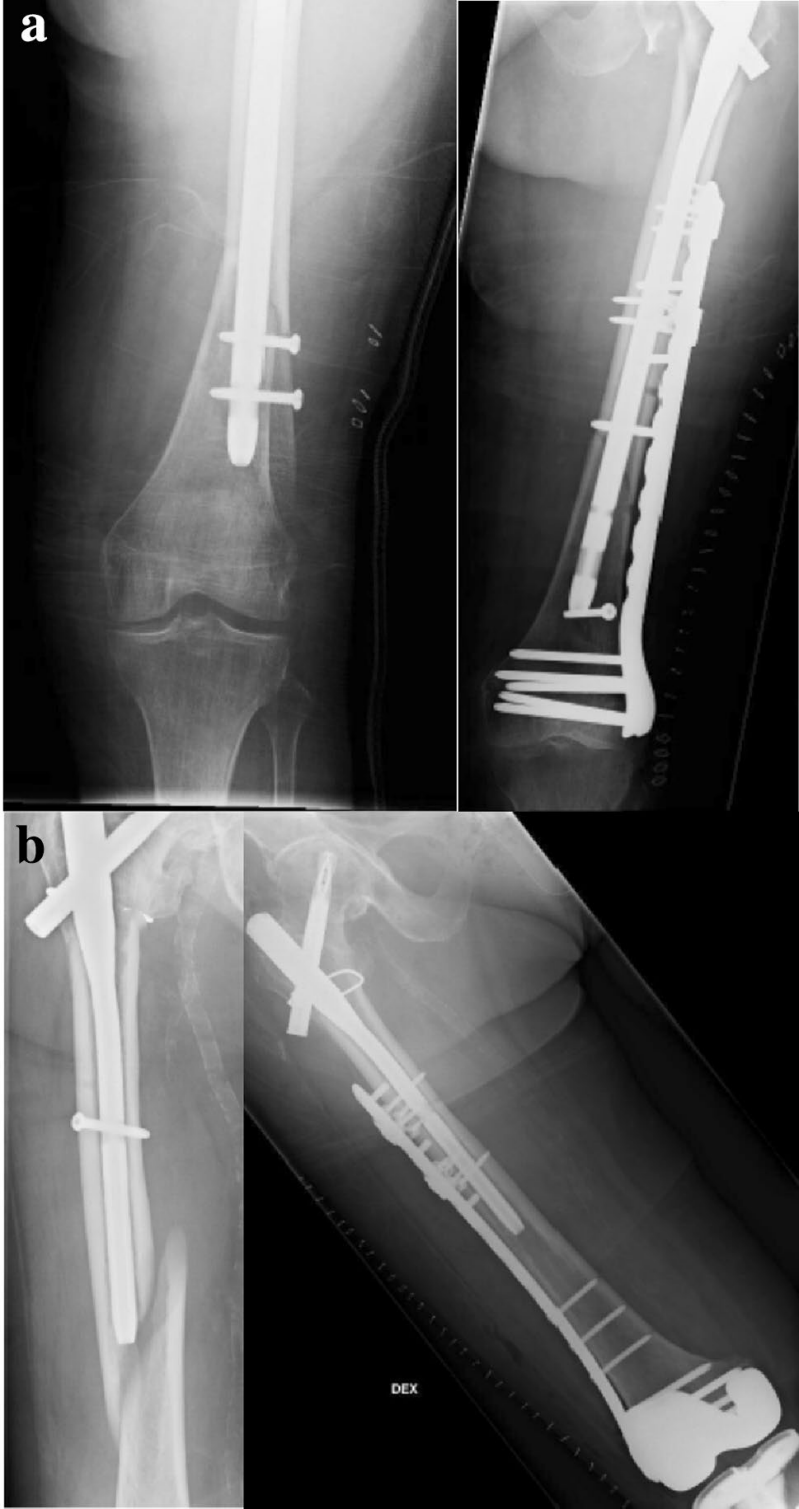
Peri-implant fracture (PIF) treated with plating without nail removal. **a** PIF of a long PFNA. **b** PIF of an intermediate-length (240 mm) PFNA

**Fig. 2:**
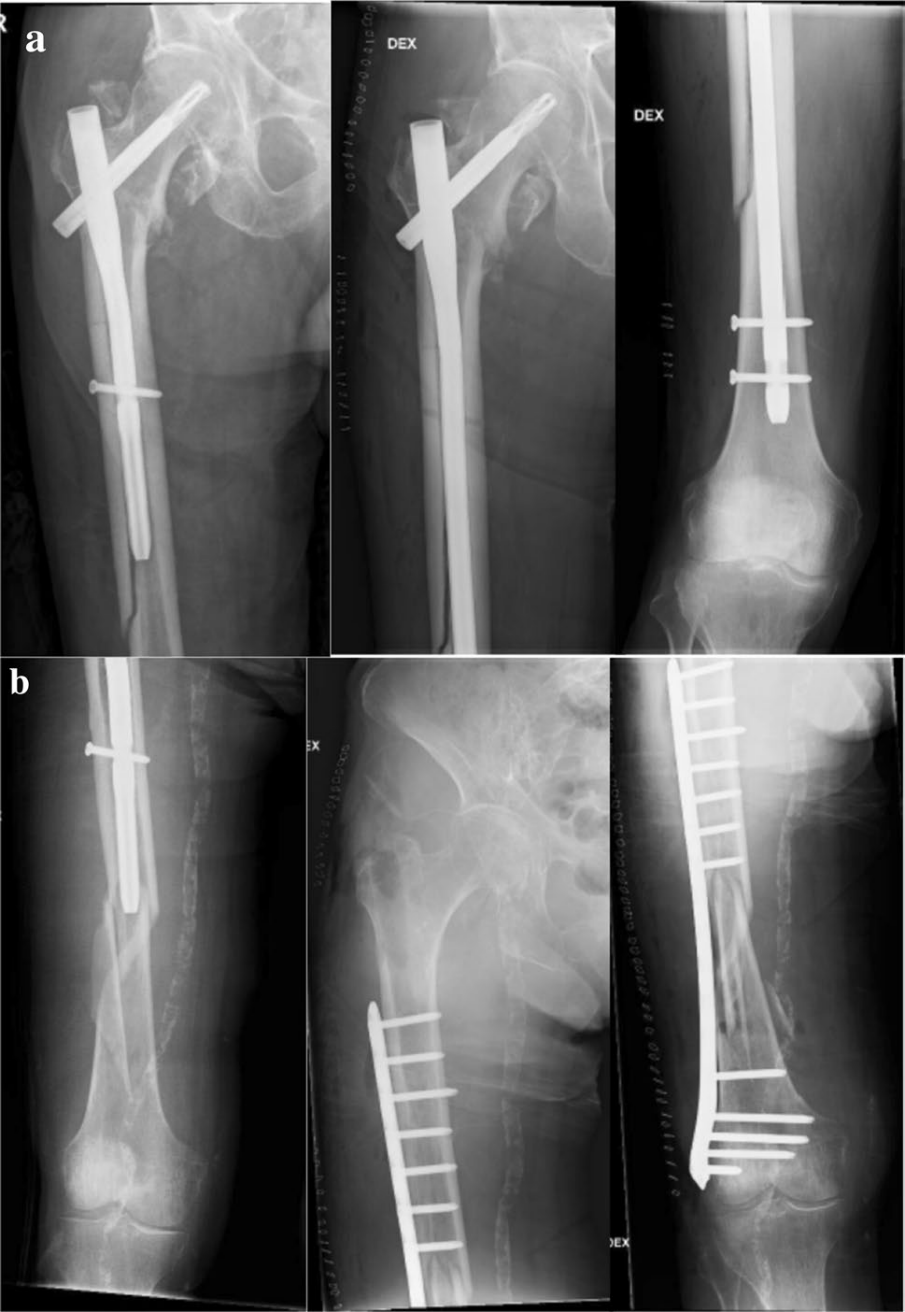
Peri-implant fracture treated with nail removal and **a** exchange nailing to a long PFNA. **b** Open reduction and internal fixation with a locking plate

The total rate of PIF was 1.4% (*n* = 14). The median time to a PIF was 102 (6–896) days after initial trochanteric fracture surgery, and the median age of patients with a PIF was 90 (61–105) years (Table 1). The most common mechanism of injury was a fall from standing height or less (79%, *n* = 11) and the remainder (21%, *n* = 3) occurred without significant injuries and were classified as stress fractures. The most common site of fracture was at the distal tip of the nail (71%, *n* = 10), whereas two patients had PIF more proximally along with the nail and two patients distal to the nail (Figs. 1 and 2). All stress fractures occurred at the tip of the IM nail (Table 2).

**Table 2:** Treatment strategy of individual patients

Patient	Age	Nail	Time to PIF^a^	Mechanism of injury	Location of fracture	Salvage treatment	Removal of IM nail	Alive at 2 years^b^
1	87	L	6	Low-energy fall	Distal tip of IM nail	Plate	No	Yes
2	82	I	33	No injury	Distal tip of IM nail	Plate	No	Yes
3	90	S	36	Low-energy fall	By the IM nail	Nail		Yes
4	89	I	43	No injury	Distal tip of IM nail	Nail		Yes
5	96	I	60	Low-energy fall	Distal tip of IM nail	Nail		No
6	75	I	67	Low-energy fall	By The IM nail	Nail		Yes
7	92	I	94	Low-energy fall	Distal tip of IM nail	Plate	No	No
8	93	I	110	Low-energy fall	Distal to the IM nail	Plate	No	No
9	92	I	122	Low-energy fall	Distal tip of IM nail	Nail		No
10	61	I	265	Low-energy fall	Distal to the IM nail	Plate	No	Yes
11	72	S	329	No injury	Distal tip of IM nail	Nail		Yes
12	91	I	496	Low-energy fall	Distal tip of IM nail	Plate	No	No
13	105	I	510	Low-energy fall	Distal tip of IM nail	Plate	Yes	No
14	72	I	896	Low-energy fall	Distal tip of IM Nail	Plate	Yes	Yes

*S* short IM nail (200 mm), *I* intermediate-length nail (240 mm), *L* long nail, *nail* exchange nailing, *plate* ORIF with a locking plate

^a^Time (days) from initial surgery to the PIF

^b^Alive at 2 years after the PIF

The rate of PIF was 2.7% (*n* = 2) for patients treated with short 200 mm nails, 1.5% (*n* = 11) with intermediate-length 240 mm nails and 0.7% (*n* = 1) with long nails, respectively. The differences between groups were not statistically significant. All post-operative intramedullary nail breakages (*n* = 3, 2.1%) occurred after treatment with a long nail. These happened at 164, 174, and 282 days from the initial fracture surgery, respectively.

**Fig. 3:**
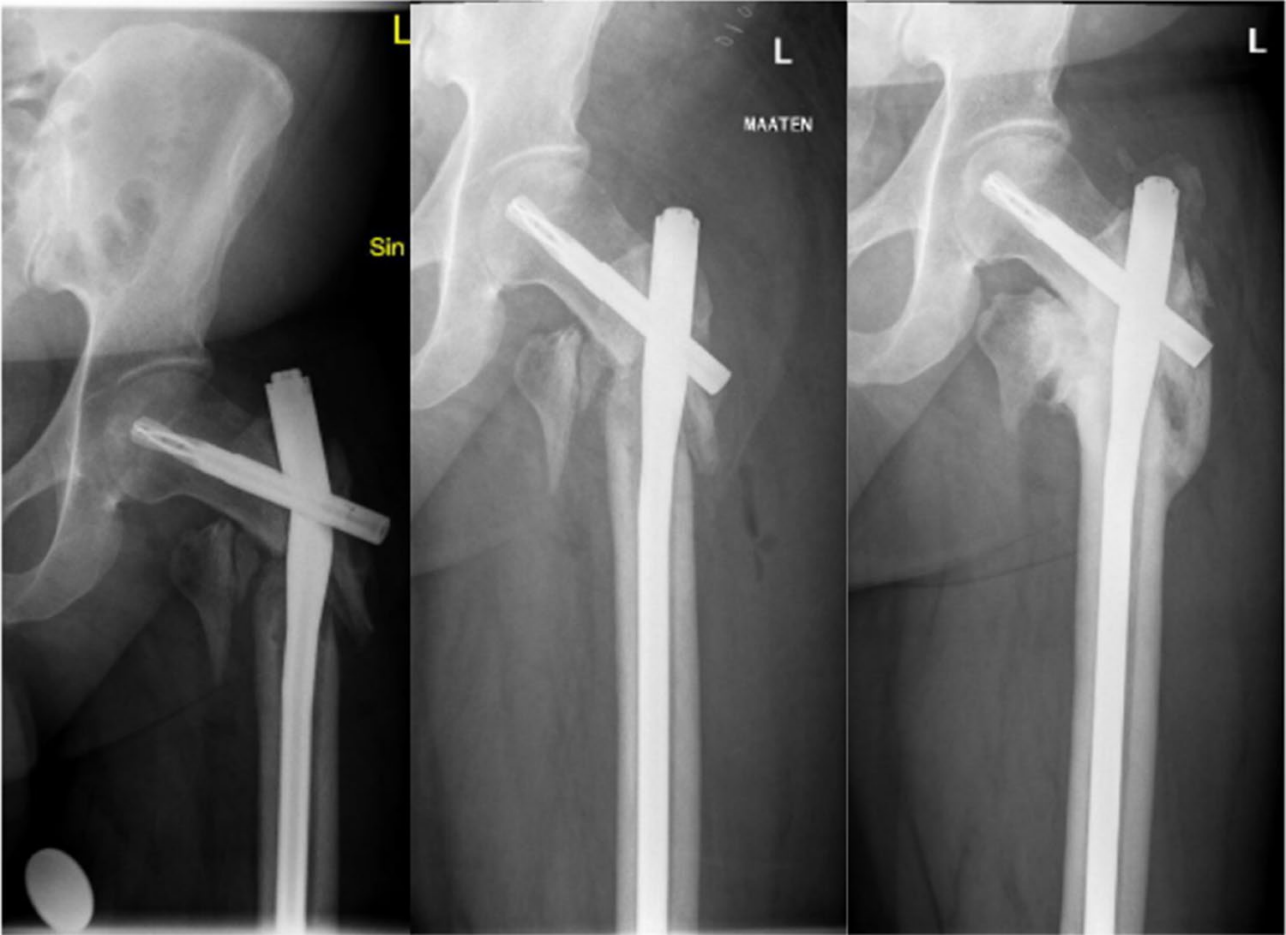
Nail breakage of a long PFNA treated with exchange nailing and autogenic bone grafting from the iliac crest

Treatment of choice for PIF was either fixation with a locking plate (*n* = 8, 57%) or exchange nailing (*n* = 6, 43%). The treatment strategy was defined based on fracture morphology and whether the initial fracture had healed. All nail breakages occurring after treatment with long nails happened by the hole for the blade (Fig. 3). These were treated with exchange nailing and with autogenic bone grafting from the iliac crest.

None of the patients needed additional surgeries for malunion, non-union, or delayed union after the initial surgery for the PIF. One patient had a post-operative surgical site infection after salvage treatment treated with a series of surgical debridements and intravenous antibiotics. The mortality after PIF was 7% at 30 and 90 days, 29% at 1 year, and 43% at 2 years. As a comparison, mortality after initial trochanteric fracture was 7% at 30 days, 14% at 90 days, 26% at 1 year, and 35% at 2 years, respectively.

The rate of blade cut-out was 2.7% (*n* = 2/74) for short nails, 1.2% (*n* = 9/728) for intermediate-length nails, and 1.3% (*n* = 2/150) for long nails. Overall, reoperation rate was 11% (*n* = 8/74) for short nails, 5.9% (*n* = 43/728) for intermediate-length nails, and 10% (*n* = 15/150) for long nails, respectively.

## Discussion

The rate of PIF (1.4%) in our study was similar to earlier reports, with 1.6–2.1% reported for PFNA and 1.7% for all cephalomedullary nails together [7, 12]. Previously, Skala-Rosenbaum et al. reported an incidence of 2.0% (*n* = 17) for PIFs in their series of 849 trochanteric fractures treated with intermediate-length (240 mm) cephalomedullary nails [13]. However, 16 of the 17 PIFs occurred in patients with no distal locking screw. Compared to our material, distinctly more PIFs occurred by the intramedullary nail which is probably caused by the absence of the distal locking screw. The patients in their series were treated by inserting distal locking screw, exchange nailing, or plating, and all the patients healed without complications. This is parallel to our study where no additional surgeries were needed due to prolonged fracture healing, although one patient suffered a post-operative infection and had multiple revision surgeries.

We found a nonsignificant trend for fewer PIFs with long nails compared to intermediate-length and short nails (0.7% vs. 1.5% vs. 2.7%). However, this advantage is negated by the risk of nail breakage with long nails (2.1%). In addition, the length of surgery and the amount of bleeding are increased when using long nails compared to short nails [14]. Fortunately, PIFs and nail breakages were rare in both groups. We do not recommend the use of long cephalomedullary nails to reduce the risk for PIF, but rather to decide the fixation method on the basis of the fracture morphology.

In recent years, four different classifications have been proposed for PIFs after intramedullary fixation of proximal femoral fractures [13, 15–17]. All classifications take into account the fracture location, whereas some include a description of the PIF morphology and/or initial fracture healing. With the validation processes still in progress, the assistance for clinicians regarding choosing the treatment remains minor.

We hypothesize that nail breakage at the hole for the collum blade with PFNA occurs similarly to nail breakage with gamma nails and is caused by delayed union [18]. Increased and prolonged stress to the weakest point of the nail makes the nail break at a certain time point. Metallurgic and electronic microscope analysis by Dragosloveanu et al. suggests that this stress might be increased because of minor malalignment of the blade from multiple insertions of the guidewire for helical blade [19]. In our study, most PIFs occurred at the tip of the nail, suggesting that the stress point at that site defines the typical site of bone to fail.

The treatment of PIFs is challenging, as many of the patients had their PIF during the recovery period from the initial surgery. The treatment strategy is dependent on fracture morphology and whether the initial fracture has healed. In our material, both exchange nailing and locking plates yielded good results. The surgeon treating these complications should be comfortable to use both techniques as well as have the capability and instrumentation for implant removal.

The main limitation of the study is its retrospective nature without patient-reported outcomes. PIFs are rare and occur to elderly patients with multiple comorbidities. For statistical analysis to be possible, a large multicenter study would be needed.

Based on these single-center study outcomes, we suggest the following treatment protocol:

(1) if the original fracture has healed and a stable fixation can be achieved by nailing, the fracture should be treated with exchange nailing to a longer intramedullary nail

(2) When the healing of the original fracture is uncertain or a stable fixation is not considered possible with a nail, fixation with a locking plate is the treatment of choice

In conclusion, PIF is a rare complication after intramedullary nailing of trochanteric fracture. Depending on fracture morphology and healing of the initial fracture, both revision surgery with exchange nailing and locking plate osteosynthesis appear to be adequate treatment options.

## References

[1] Gromov K, Brix M, Kallemose T, Troelsen A (2014). Early results and future challenges of the danish fracture database. Dan Med J.

[2] Nyholm AM, Palm H, Malchau H, Troelsen A, Gromov K (2016). Lacking evidence for performance of implants used for proximal femoral fractures—a systematic review. Injury.

[3] Socci AR, Casemyr NE, Leslie MP, Baumgaertner MR (2017). Implant options for the treatment of intertrochanteric fractures of the hip: rationale, evidence, and recommendations. Bone Jt J.

[4] Nie B, Wu D, Yang Z, Liu Q (2017). Comparison of intramedullary fixation and arthroplasty for the treatment of intertrochanteric hip fractures in the elderly: a meta-analysis. Medicine (Baltimore).

[5] Sreekanta A, Eardley WG, Parker MJ (2019). Surgical interventions for treating extracapsular hip fractures in adults: A network meta-analysis. Cochrane Libr.

[6] Werner BC, Fashandi AH, Gwathmey FW, Yarboro SR (2015). Trends in the management of intertrochanteric femur fractures in the united states 2005–2011. Hip Int.

[7] Norris R, Bhattacharjee D, Parker MJ (2011). Occurrence of secondary fracture around intramedullary nails used for trochanteric hip fractures: A systematic review of 13,568 patients. Injury.

[8] Saul D, Riekenberg J, Ammon JC, Hoffmann DB, Sehmisch S (2019). Hip fractures: therapy, timing, and complication spectrum. Orthop Surg.

[9] Flikweert ER, Wendt KW, Diercks RL (2017). Complications after hip fracture surgery: are they preventable?. Eur J Trauma Emerg Surg.

[10] Koval KJ (2007). Intramedullary nailing of proximal femur fractures. Am J Orthop (Belle Mead, NJ).

[11] Buckley RE, Moran CG, Apivatthakakul T (2017). AO principles of fracture management.

[12] Müller F, Galler M, Zellner M, Bäuml C, Marzouk A, Füchtmeier B (2016). Peri-implant femoral fractures: the risk is more than three times higher within PFN compared with DHS. Injury.

[13] Skála-Rosenbaum J, Džupa V, Bartoška R, Douša P, Waldauf P, Krbec M (2016). Distal locking in short hip nails: cause or prevention of peri-implant fractures?. Injury.

[14] Dunn J, Kusnezov N, Bader J, Waterman B, Orr J, Belmont P (2016). Long versus short cephalomedullary nail for trochanteric femur fractures (OTA 31–A1, A2 and A3): a systematic review. J Orthop Traumatol.

[15] Toro G, Moretti A, Ambrosio D (2021). Fractures around trochanteric nails: the “Vergilius classification system”. Adv Orthop.

[16] Videla-Cés M, Sales-Pérez J, Sánchez-Navés R, Romero-Pijoan E, Videla S (2019). Proposal for the classification of peri-implant femoral fractures: retrospective cohort study. Injury.

[17] Chan L, Gardner A, Wong M, Chua K, Kwek E (2018). Non-prosthetic peri-implant fractures: classification, management and outcomes. Arch Orthop Trauma Surg.

[18] Johnson NA, Uzoigwe C, Venkatesan M (2017). Risk factors for intramedullary nail breakage in proximal femoral fractures: a 10-year retrospective review. Ann R Coll Surg Engl.

[19] Dragosloveanu Ş, Dragosloveanu CDM, Stanca HT, Cotor DC, Dragosloveanu CI, Stoica CI (2020). A new perspective toward failure of gamma nail systems. Exp Ther Med.

